# Oxidative Stress and Multi-Organel Damage Induced by Two Novel Phytocannabinoids, CBDB and CBDP, in Breast Cancer Cells

**DOI:** 10.3390/molecules26185576

**Published:** 2021-09-14

**Authors:** Maria Salbini, Alessandra Quarta, Fabiana Russo, Anna Maria Giudetti, Cinzia Citti, Giuseppe Cannazza, Giuseppe Gigli, Daniele Vergara, Antonio Gaballo

**Affiliations:** 1 CNR Nanotec, Institute of Nanotechnology, Via Monteroni, 73100 Lecce, Italy; maria.salbini@nanotec.cnr.it (M.S.); alessandra.quarta@nanotec.cnr.it (A.Q.); cinzia.citti@nanotec.cnr.it (C.C.); giuseppe.cannazza@unimore.it (G.C.); giuseppe.gigli@unisalento.it (G.G.); 2 Clinical and Experimental Medicine PhD Program, University of Modena and Reggio Emilia, 41125 Modena, Italy; fabiana.russo@unimore.it; 3 Department of Biological and Environmental Sciences and Technologies, University of Salento, Via Monteroni, 73100 Lecce, Italy; anna.giudetti@unisalento.it (A.M.G.); daniele.vergara@unisalento.it (D.V.); 4 Department of Life Sciences, University of Modena and Reggio Emilia, Via G. Campi 103, 41125 Modena, Italy; 5 Dipartimento di Matematica e Fisica E. de Giorgi, Università Del Salento, 73100 Lecce, Italy

**Keywords:** MCF-7, cannabidiol, ROS, oxidative stress, autophagy, altered mitochondria, cytoplasmic vacuole, MAGL, MJN110

## Abstract

Over the last few years, much attention has been paid to phytocannabinoids derived from Cannabis for their therapeutic potential. Δ^9^-tetrahydrocannabinol (Δ^9^-THC) and cannabidiol (CBD) are the most abundant compounds of the *Cannabis sativa L.* plant. Recently, novel phytocannabinoids, such as cannabidibutol (CBDB) and cannabidiphorol (CBDP), have been discovered. These new molecules exhibit the same terpenophenolic core of CBD and differ only for the length of the alkyl side chain. Roles of CBD homologs in physiological and pathological processes are emerging but the exact molecular mechanisms remain to be fully elucidated. Here, we investigated the biological effects of the newly discovered CBDB or CBDP, compared to the well-known natural and synthetic CBD (nat CBD and syn CBD) in human breast carcinoma cells that express CB receptors. In detail, our data demonstrated that the treatment of cells with the novel phytocannabinoids affects cell viability, increases the production of reactive oxygen species (ROS) and activates cellular pathways related to ROS signaling, as already demonstrated for natural CBD. Moreover, we observed that the biological activity is significantly increased upon combining CBD homologs with drugs that inhibit the activity of enzymes involved in the metabolism of endocannabinoids, such as the monoacylglycerol lipase (MAGL) inhibitor, or with drugs that induces the activation of cellular stress pathways, such as the phorbol ester 12-myristate 13-acetate (PMA).

## 1. Introduction

Cannabinoids include a wide group of organic molecules, including those that are physiologically produced in the human body, called endocannabinoids, those extracted primarily from the *Cannabis sativa L.* plant, named phytocannabinoids, and synthetic cannabinoids [1]. Recently, phytocannabinoids, in particular cannabidiol (CBD) and Δ^9^-tetrahydrocannabinol (THC), have been widely exploited in several research and clinical fields [2]. In the last few years, the homologous series of CBD has been expanded by the isolation in a medicinal cannabis variety of novel phytocannabinoids such as cannabigerol (CBG), cannabichromene (CBC), cannabinol (CBN) and cannabidivarin (CBDV) [3,4,5,6,7,8]. Although these compounds have similar chemical structures, they can elicit different biological actions. Phytocannabinoids demonstrated a selective anti-cancer activity in many cancer cell lines, by affecting cell proliferation, differentiation, and death [9]. In light of this, a possible role as an adjuvant in many cancer therapies has been proposed. Recently, it was found that the co-administration of cannabinoids with chemotherapeutic drugs enhanced their efficiency, especially in chemotherapy-refractory tumors [10,11]. Phytocannabinoids may act via dependent and/or independent cannabinoid receptor mechanisms [12,13]. CB1 and CB2 are the first cannabinoids receptors described. These are G-protein coupled receptors (GPCR) that can be activated by endogenous and exogenous cannabinoids. More recently, studies have shown that cannabinoids can activate other receptors, i.e., GPR55, TRPM8 as well as other ion channels of the transient receptor potential superfamily as vanilloid type 1–4 (TRPV1, TRPV2, TRPV3 and TRPV4) [12,14,15,16,17]. In the context of breast cancer, the biological role of CBD in the regulation of epithelial tumor pathophysiology has clearly emerged [18,19]. Tumor cells express CBD receptors and respond to these molecules by activating specific signaling pathways. For instance, Shrivastava A. et al., [20] described that in breast cancer cells, cannabidiol induces the generation of reactive oxygen species (ROS), endoplasmic reticulum (ER)-stress and subsequently the activation of autophagic processes. Here, we investigated in vitro the biological effects of CBD homologs, the newly discovered CBDB or CBDP, in breast cancer models. We observed that CBD homologs induced changes in ROS levels and cellular processes related to ROS signaling. Furthermore, CBD homologs affected the morphology and functionality of several cell structures, such as mitochondria and ER, as already demonstrated for natural CBD [21]. The combination of CBD homologs with drugs that inhibit the activity of enzymes involved in the metabolism of endocannabinoids, such as the monoacylglycerol lipase (MAGL), or with drugs that induce the activation of cellular stress pathways, such as the phorbol ester 12-myristate 13-acetate (PMA), is associated with an extensive vacuolization, hyper-increased ROS levels, and multiple alterations in organelles structure. In summary, we investigated the biological effects of two novel CBD homologs in breast cancer cells and reported, for the first time, the activation of catastrophic processes after the combination of CBDB or CBDP with drugs that modulate the metabolism of endocannabinoids or regulate the activation of specific protein kinases.

## 2. Results

### 2.1. Synthesis and Characterization of Phytocannabinoids

**Figure 1:**
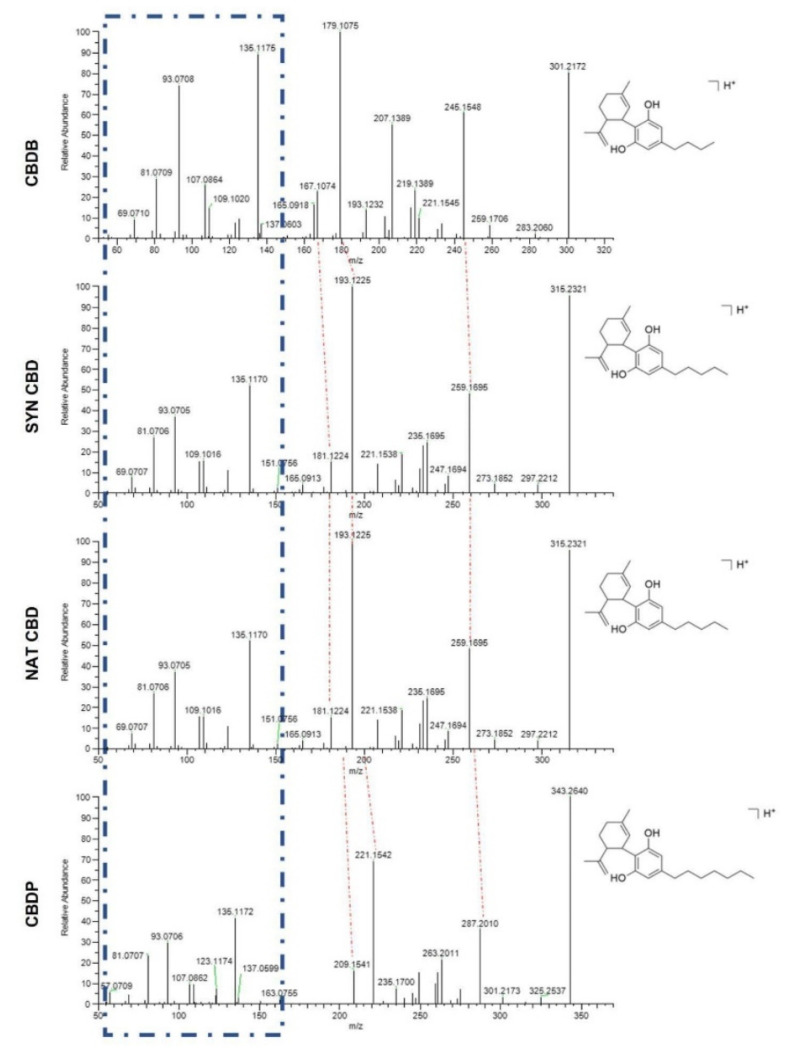
HPLC-HRMS analysis in positive ionization mode of the four CBD homologs under investigation. Blue dotted lines highlight the common pattern, while red dotted lines show the difference in the fragments due to the number of methylene units on the alkyl side chain.

CBD of both synthetic and natural origin, syn CBD and nat CBD respectively, are commercially available, while cannabidibutol (CBDB) and cannabidiphorol (CBDP) have been recently discovered and isolated from the Italian medicinal variety FM2 of *Cannabis sativa* L. [4,6,7]. In these works, the stereoselective synthesis of the same compounds allowed absolute stereochemistry by comparison of their spectroscopic and optical properties [4,6,7]. Nat CBD and syn CBD are identical molecules, therefore we expect to see no difference in their biological behavior. However, high-performance liquid chromatography coupled to high-resolution mass spectrometry (HPLC-HRMS) analyses has recently shown that nat CBD contains small amounts (0.1–0.5%, *w*/*w*) of two impurities corresponding to CBD propyl and butyl homologs, cannabidivarin (CBDV) and CBDB respectively [4]. The CBD homologs under investigation in the present work differ only by the length of the alkyl side chain on the resorcinyl moiety. In particular, CBDB has a linear C4 (butyl) side chain, both nat CBD and syn CBD have a C5 (pentyl) chain, and CBDP has a C7 (heptyl) chain (Figure 1). The four cannabinoids present similar physicochemical and spectroscopic properties: UV and FT-IR spectra are perfectly superimposable, while Nuclear Magnetic Resonance (NMR) spectroscopy shows a difference only in the signal corresponding to the alkyl chain; HRMS data highlight a perfect match of all *m*/*z* of the fragments with the difference only in the number of methylene units [4,6,7]. The length of the alkyl side chain could dramatically affect the affinity for the biological targets as it has been demonstrated for THC, a CBD isomer [22].

### 2.2. CBD Homologs Treatment Significantly Decreased Cancer Cell Viability

**Figure 2:**
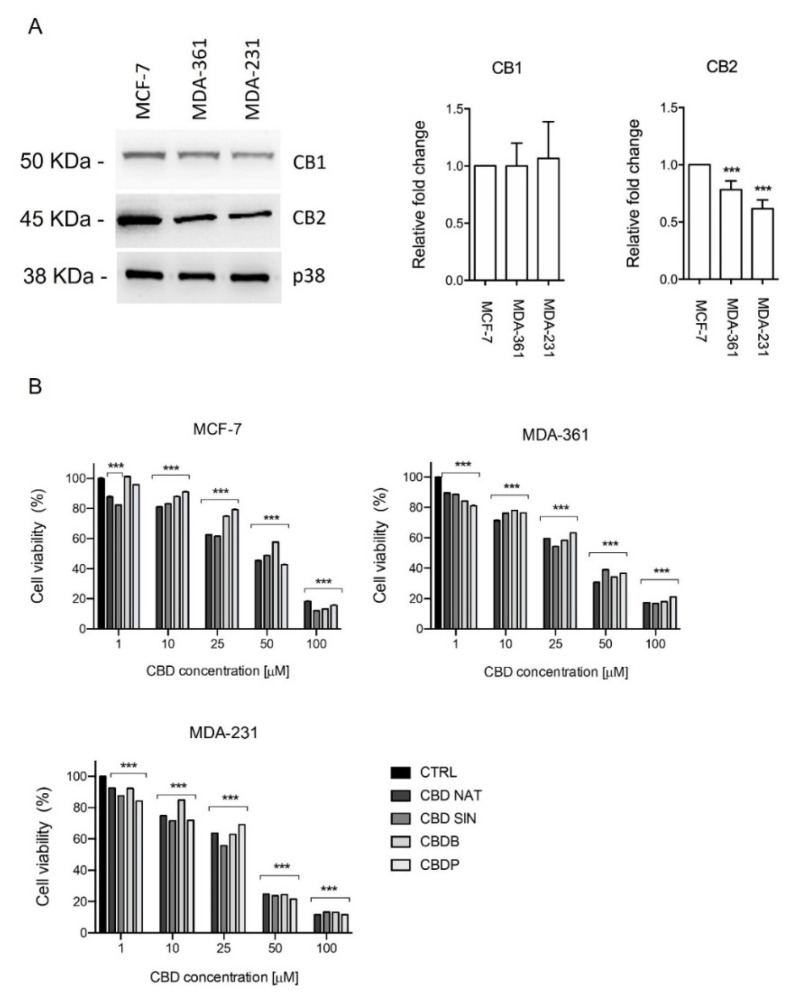
(**A**) Immunoblot analysis of CB1 and CB2 receptors in MCF-7, MDA-MB-361 and MDA-MB-231 breast cancer cell lines, p38 was used as a loading control. Densitometric quantification of the band intensity was normalized to p38 levels using ImageJ. *p*-value *** < 0.001 by *t*-test. Effect of cannabinoid treatment on cell viability: (**B**) MCF-7, MDA-MB-361 and MDA-MB-231 cells. All the cell lines were treated with different concentrations of nat CBD, syn CBD, CBDB and CBDP for 24 h; the cell viability was measured using the MTT assay. Values are the mean ± SD. *** *p* < 0.0001 compared with controls.

The biological effects of phytocannabinoids are thought to be due to their affinity to CB1 and CB2 receptors. The expression of these proteins was investigated in vitro using a panel of breast cancer cell models (estrogen and progesterone receptor positive: MCF-7, MDA-MB-361; and estrogen and progesterone receptor negative: MDA-MB-231). Qualitative analysis of cannabinoid targets in vitro showed that the three breast cancer cells express both CB1 and CB2 receptors (Figure 2A).

Subsequently, before investigating the specific activity of the CBD homologs on cancer cell lines, we screened their cytotoxicity on two different epithelial and one mesenchymal breast cancer cell lines namely MCF-7, MDA-MB-361 and MDA-MB-231, using the metabolism-dependent MTT viability assay. Cells were treated with various concentrations (from 1 to 100 µM) of nat CBD, syn CBD, CBDB and CBDP, for 24 h. All CBD homologs inhibited the viability of breast cell lines with lower activity in MCF-7 and MDA-MB-361 cells, while the higher effect was observed in MDA-MB-231 (Figure 2B). Table 1 summarizes the IC_50_ values for all the tested molecules. The striking behavior of CBD homologs treatments was the occurrence of massive cytoplasmic vacuolation in the epithelial MCF-7 cells (Figure S1). Vacuoles already appeared after 2 h of treatment and their number and size increased after 24 h. The same changes were less pronounced in mesenchymal MDA-MB-231 cells, while in the epithelial MDA-MB-361 cells no vacuolation occurred (Figure S1). This preliminary analysis evidenced that MCF-7 cells while preserving their viability, displayed macroscopic morphological alterations upon CBD homologs administration. To shed light on the possible intracellular effects, we focused on the comparative analyis of the four CBD homologs on this cellular model. Indeed, while nat CBD and syn CBD are identical and quite known molecules, the cellular effects of the newly isolated CBDB and CBDP are not investigated yet. The nat and syn CBD are used as reference molecules. All the experimental data presented in the following sections have been performed using the four CBD homologs, although particular attention will be dedicated to the two new ones, at a concentration of 10 μM. In line with the literature [18,23,24], this is a working concentration that induces biological responses without dramatic effects on cell viability.

### 2.3. CBD Homologs Treatment Significantly Increased ROS Production in MCF-7 Cells

**Figure 3:**
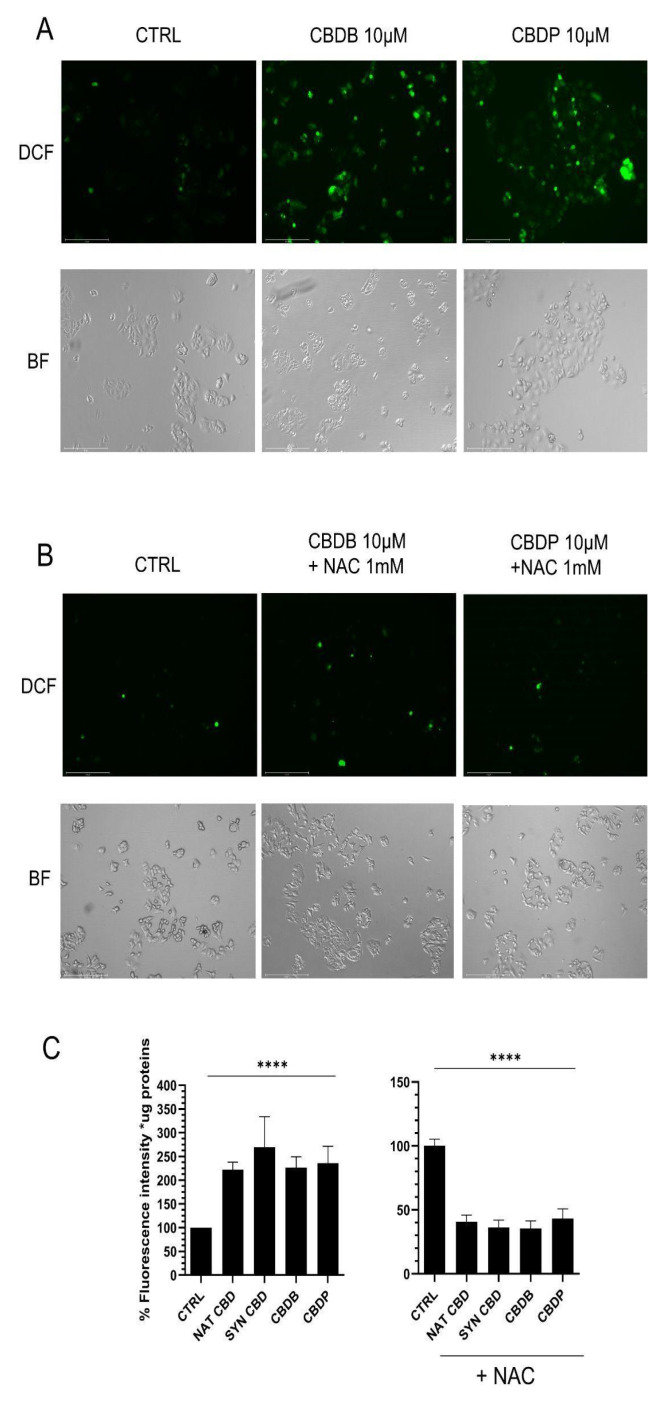
Representative fluorescent images of 2′,7′-dichlorodihydrofluorescein diacetate (DCFH-DA) staining in MCF-7 cells and its corresponding image in bright field (BF). (**A**) MCF-7 cells treated for 24 h with vehicle (CTRL), CBDB or CBDP (10 µM). (**B**) MCF-7 cells treated with CBDB or CBDP (10 µM) and 1 mM N-acetylcysteine (NAC), for 24 h. All images were taken by Evos m7000 fluorescence microscope objective 10x, scale bar 275 µm. (**C**) Fluorescence intensity quantification by a fluorescence microplate reader for DCFH staining in MCF-7 cells control and after CBD, and NAC+ CBD homologs, after 24 h. Values are the mean ± SD. **** *p* < 0.0001 compared with controls.

An increase in the oxidative stress has been detected in cancer cells after CBD treatment due to a massive increase of ROS production that leads to the activation of apoptosis and autophagy [14,16,17]. Starting from these data, we analyzed ROS levels in MCF-7 cells treated with 10 µM nat CBD, syn CBD, CBDB and CBDP for 24 h using the 2′,7′-dichlorodihydrofluorescein diacetate (DCFH-DA) staining. The DCFH-DA probe is nonfluorescent in its initial form, but it can be easily oxidized by intracellular ROS, leading to the formation of the fluorescent product dichlorofluorescein (DCF). The antioxidant N-acetyl cysteine (NAC) was used to counteract ROS production. As shown in Figure 3A,C, a significant increase in intracellular ROS levels was observed in MCF-7 cells treated with CBD homologs. On the contrary, NAC treatment in association with CBD homologs reverted the relative fluorescence intensity to a value lower than control, due to a remarkable ROS scavenging effect (Figure 3B,C).

### 2.4. CBD Homologs Treatment Altered Mitochondria and ER in MCF-7 Cells

**Figure 4:**
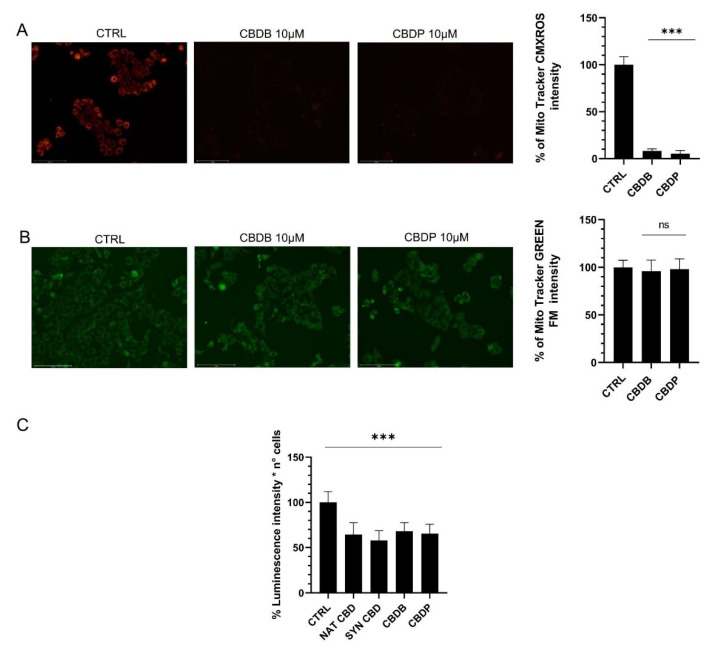
Representative fluorescent images of the mitochondrial function. MCF-7 cells were treated with vehicle (CTRL), CBDB and CBDP (10 µM) for 24 h. (**A**) Cells were stained with the membrane potential-dependent dye MitoTracker CMXRos. (**B**) Cells were stained with the MitoTracker Green FM. Both fluorescent markers were imaged with an Evos m7000 fluorescence microscope. Scale bar 150 μm), objective 20x. (**C**) After treatments, the cellular ATP level was measured with the CellTiter-Glo 2.0 cell viability luminescence assay. Values are the mean ± SD. *** *p* < 0.001 compared to controls; ns not significant.

To establish if there is a correlation between ROS production and impaired mitochondrial functions, we used two mitochondria stains, Mitotracker Red and MitoTracker Green. The first is an indicator of mitochondrial membrane potential that selectively stains active mitochondria, while MitoTracker Green allows the detection of the mitochondrial morphology and mass. When MCF-7 cells were exposed to 10 μM CBDB or CBDP for 24 h, the Mitotracker red fluorescence intensity dramatically dropped as compared to the control (Figure 4A). Similar results were obtained with nat CBD and syn CBD (data not shown). On the other hand, the MitoTracker Green fluorescence signal remained similar in control and treated cells (Figure 4B). These observations suggest that the treatment with CBD homologs leads to an alteration of the mitochondrial functionality without an appreciable decrease of the mitochondrial mass. The loss of the mitochondrial function was also accompanied by a substantial decrease of ATP, whose levels dropped by approximately 35–40% compared to controls in MCF-7 cells treated with all CBD homologs (Figure 4C).

**Figure 5:**
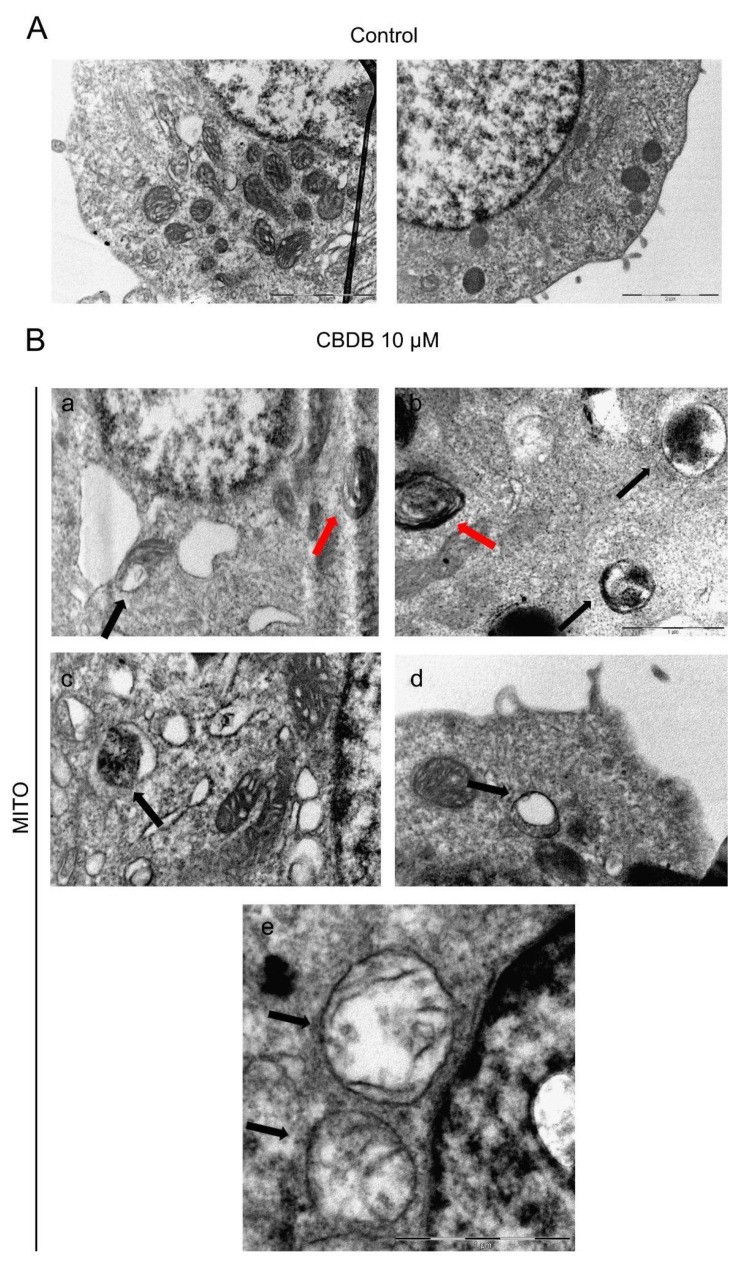
Representative TEM images of cell morphology. (**A**) MCF-7 control cells (treated with DMSO as a vehicle). Scale bar 2 μm. (**B**) MCF-7 cells exposed to CBDB or CBDP (10 µM) for 24 h. Mitochondria with dilated cristae (panel **a**,**b**, red arrows); swollen mitochondria with crests in the process of breaking (panel **a**–**e**; black arrows). Scale bar 1 μm.

To further elucidate the effects of CBDB and CBDP treatment in MCF-7 cells, ultrastructural analysis employing transmission electron microscopy (TEM) was performed. TEM imaging revealed multiple damages to cellular organelles. Interestingly, as already observed by optical imaging, cytoplasmic vacuolations were detected. In addition, mitochondria with altered morphology, such as rounded and rod-like mitochondria and dilated cristae were distinguished (Figure 5B, panel a, red arrow). Figure 5B (panel a–e, black arrows) displays swollen mitochondria, with broken cristae and decreased electron density of the lumen.

**Figure 6:**
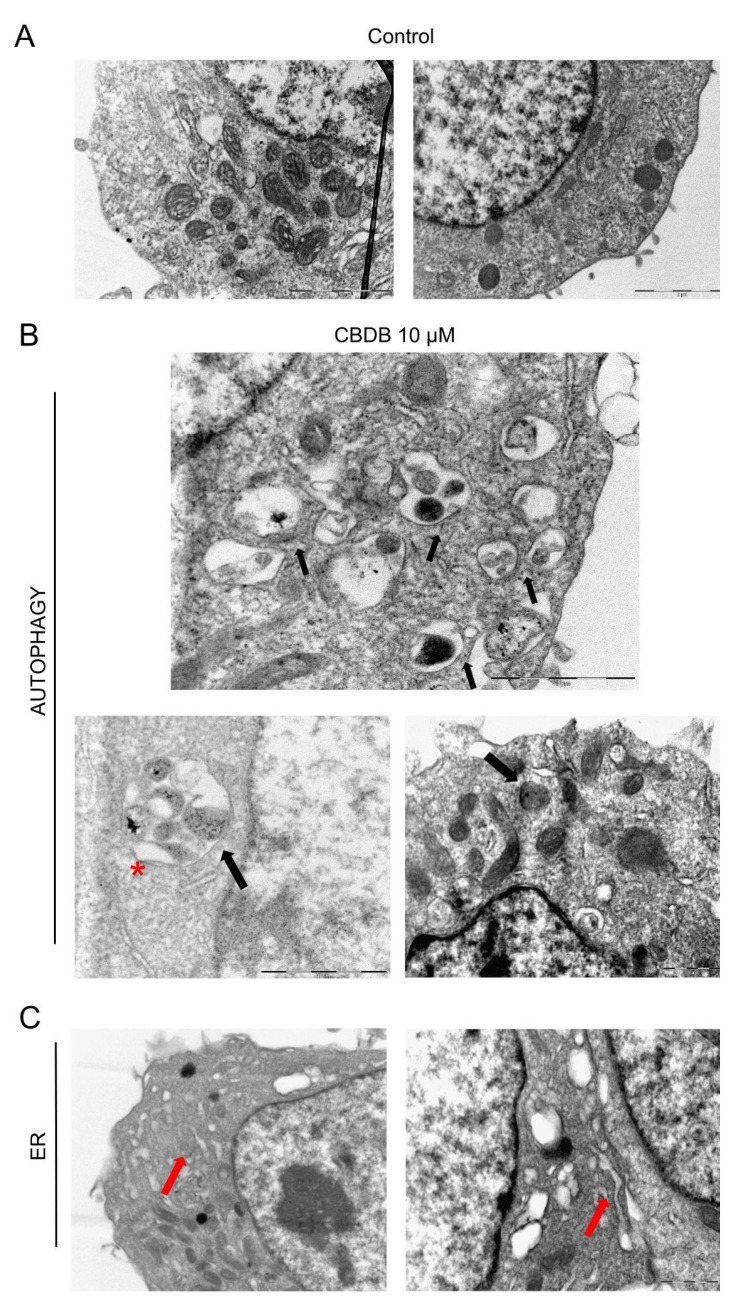
Representative TEM images of cell morphology. (**A**) MCF-7 control cells (treated with DMSO, as vehicle). Scale bar 2 μm. (**B**,**C**) MCF-7 cells fixed exposed to CBDB or CBDP (10 µM) for 24 h; (**B**) double-membrane autophagic vacuoles red asterisk, autophagosomes red arrows, (**C**) outstretched ER black arrows.

**Figure 7:**
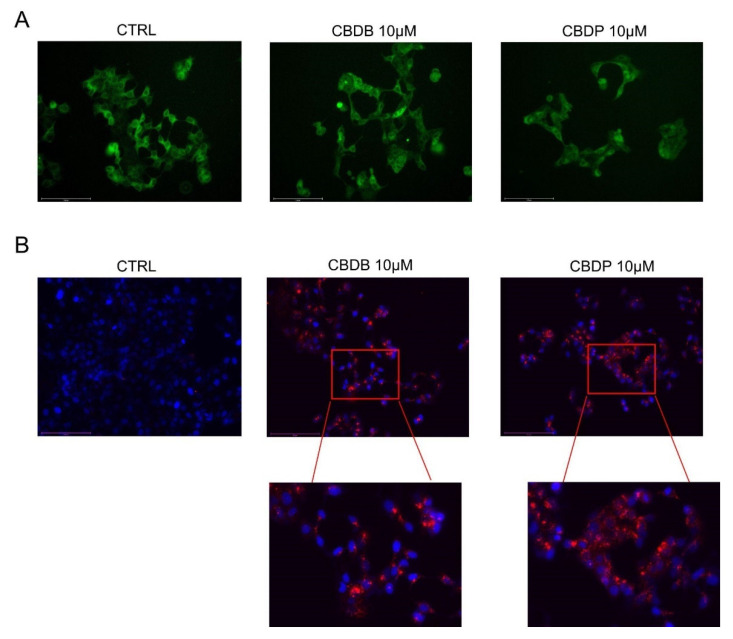
Representative fluorescent images of organelle structures. MCF-7 cells were treated with vehicle (CTRL) or CBDB and CBDP (10 µM) for 24 h. (**A**) Cells were stained with ER TrackerTM to visualize endoplasmic reticulum membranes. (**B**) Cells were stained with Hoechst (0.5 mg/mL) and Lysotracker Red DND-99 (100 nM) according to the manufacturer’s recommendations and imaged with an Evos m7000 fluorescence microscope. Scale bar 150 μm, objective 20×.

TEM imaging also revealed multiple dramatic changes in other cellular compartments after CBDB or CBDP (10 μM) exposure. Figure 6B shows the accumulation of double-membrane (Figure 6B, red asterisk) autophagic vacuoles containing cellular organelles and electron-dense material (Figure 6B, black arrows). Figure 6C shows that the ER also appears enlarged and disassembled (red arrows). These data were confirmed by ER staining with ER tracker which exhibited an increased fluorescence signal surrounding the vacuole membrane, suggesting that some cytoplasmic vacuolations can also arise from the ER membranes, due to a massive ER stress (Figure 7A).

The presence of late autophagic vesicles in cells treated with CBD homologs was also indirectly confirmed through staining with LysoTracker Red, as it is selective for acidic organelles. Cell nuclei were stained with Hoechst 33342. MCF-7 cells treated with CBD homologs displayed a higher number of lysosomes, compared to the control (Figure 7B). This observation suggests a massive presence/accumulation of autophagolysosomes.

### 2.5. Biological Effects of CBD Homologs in Combination with Either an MAGL Inhibitor 2,5-Dioxopyrrolidin-1-yl 4-(bis(4-chlorophenyl)methyl)piperazine-1-carboxylate (MJN110) and Phorbol Ester 12-Myristate 13-Acetate (PMA)

**Figure 8:**
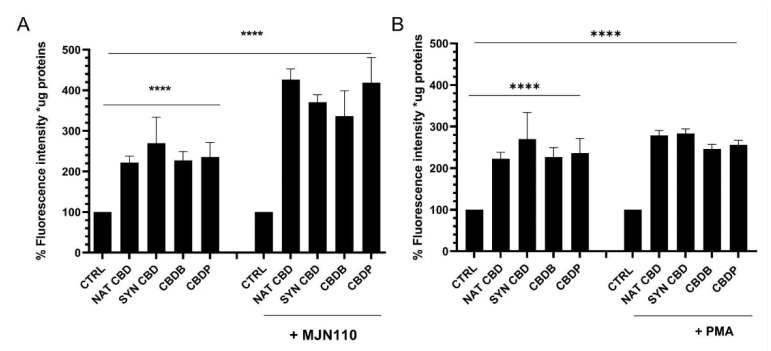
(**A**) Intensity quantification by a fluorescence microplate reader for DCFH-DA staining in MCF-7 cells after 24 h treatment with CBD (10 μM), and MJN110 (1 μM) + CBD homologs. Values are the mean ± SD. **** *p* < 0.0001 compared with controls. (**B**) Intensity quantification by a fluorescence microplate reader for DCFH-DA staining in MCF-7 cells, including control samples (DMSO) and cells treated for 24 h with CBD (10 μM), and PMA (100 nM) + CBD homologs. Values are the mean ± SD. **** *p* < 0.0001 compared with controls.

**Figure 9:**
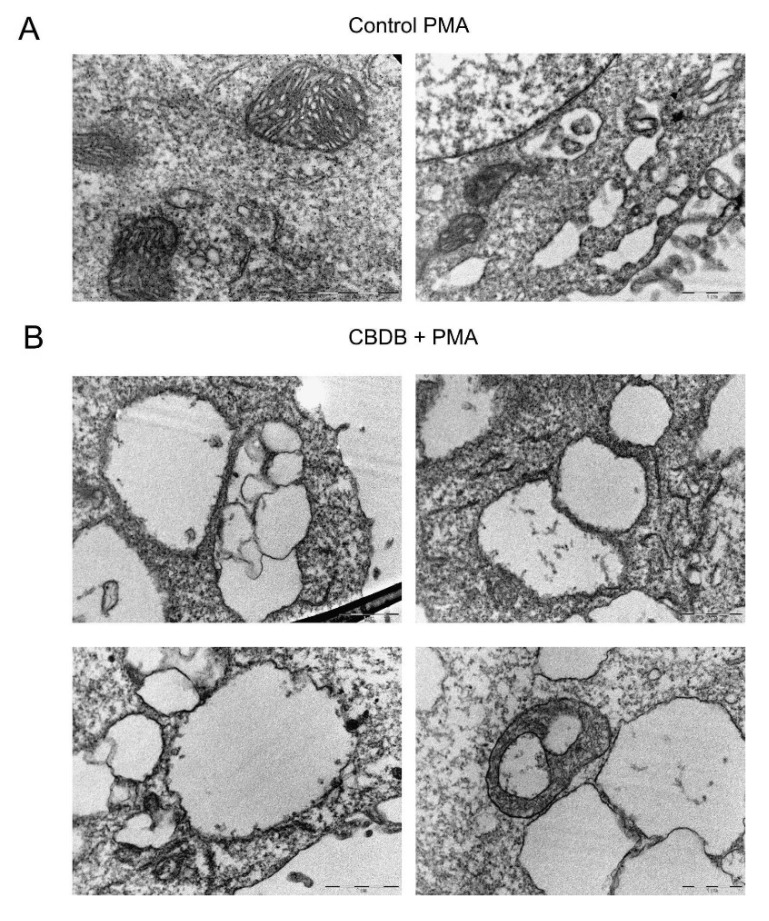
Representative TEM images of cell morphology. (**A**) MCF-7 cells were treated with vehicle (DMSO) and PMA (100 nM), showing normal cell organelles. Scale bar 2 μm. (**B**) MCF-7 cells were incubated with CBDB + PMA (100 nM), highlighting the presence of cytoplasmic vacuolations related to disassembled ER and mitochondria. Scale bar 1 μm.

We analyzed the effect of CBD homologs on MCF-7 cells in combination with MJN110 (2,5-dioxopyrrolidin-1-yl 4-(bis(4-chlorophenyl) methyl)piperazine-1-carboxylate), a MAGL inhibitor. MAGL is an enzyme that, in addition to its ability to hydrolyse monoglycerides, has been shown to have a role in endocannabinoid catabolism. Indeed it can hydrolyse 2-arachidonoyl glycerol into arachidonic acid [25]. In particular, the treatment with MJN110 (1 μM) in combination with CBDB or CBDP induced severe cellular morphological changes, with an increase of about 30% in the number of cytoplasmic vacuolations, as compared to the treatments with the individual molecules (i.e., CBD homologs or MJN110) (Figure S2A,B). A similar effect, with a more prominent increase in cytoplasmic vacuolations (about 60%) was observed by treating the cells with CBDB or CBDP in combination with PMA (100 nM), a drug that has been shown to induce the activation of different cellular stress pathways [26,27] (Figure S2A,C). Furthermore, we observed a rise in ROS levels in MCF-7 cells treated with both MJN110 and PMA (Figure 8). Dramatic changes in cell morphology, as well as in organelle structures, were confirmed by TEM imaging of MCF-7 cells treated with CBDB or CBDP in the presence of MJN110 (Figure S3). In addition, double-membrane vacuoles, containing degrading materials, were much more increased in MJN110-CBD homologs treated cells, as compared to their control. Treatment with PMA, in the same way, highlighted the presence of cytoplasmic vacuolations related to disassembled ER and mitochondria (Figure 9). The dramatic changes in cell morphology, as well as in organelle structures, appeared more catastrophic than those previously observed after the treatments with CBDB or CBDP alone or with MJN110 or PMA alone. As we mentioned before, the treatment of MCF-7 cells with CBDB or CBDP also induced ER and/or mitochondria dilation. Notably, after the combined exposure to CBD homologs and the two drugs, the morphological changes at the ER level and the quantitative increase of the lysosomes were even more remarkable (Figures S4 and S5).

## 3. Discussion

Here we conducted an in vitro study to investigate the biological effects of the newly discovered CBDB or CBDP, compared to the well-known nat CBD and syn CBD. As already mentioned, the CBD homologs under investigation differ only by the length of the alkyl side chain on the resorcinyl moiety. CBDB has a linear C4 (butyl) side chain, both nat CBD and syn CBD have a C5 (pentyl) chain, and CBDP has a C7 (heptyl) chain. The length of the alkyl side chain may affect the affinity for some biological targets as it has been demonstrated for THC, which is a CBD isomer [22]. Furthermore, due to their implication in the control of cell growth and death, cannabinoids have been proposed as a new adjuvant in cancer therapy of various malignancies, such as prostate and breast cancer [17,28]. As already described, phytocannabinoids may act via dependent and/or independent cannabinoid receptor mechanisms [13,15]. In the context of breast cancer, the biological effect of CBD in the regulation of epithelial tumor pathophysiology has clearly emerged [18,19] and is thought to be due to multiple molecular targets including the CB1 and CB2 receptors. The expression of these proteins was investigated in vitro using a panel of breast cancer cell models (estrogen and progesterone receptor positive: MCF-7, MDA-MB-361; and estrogen and progesterone receptor negative: MDA-MB-231). Qualitative analysis of cannabinoid targets in vitro showed that CB1 and CB2 were expressed in all breast cancer cells confirming already published data [29] (Figure 2A).

Within this frame, the cellular effects of the newly isolated CBDB or CBDP are not investigated yet. First we tested the antiproliferative effects of the four CBD homologs, using the metabolism-dependent MTT viability assay. For this aim, we treated MCF-7, MDA-MB-361 and MDA-MB-231 (Figure 2B) with different concentrations (1–100 µM) of nat CBD, syn CBD, CBDB and CBDP, for 24 h.

All tested molecules showed an anti-proliferative effect in triple negative, estrogen receptor positive (ER+) and progesteron receptor positive (PR+) breast tumor cell lines. More in detail, MCF-7 and MDA-MB-361 showed a lower sensitivity to CBDB or CBDP treatment in comparison to MDA-MB-231, as described in Table 1. Additional investigations are needed to gain further insights into the cellular mechanisms and the role of ER/PR signalling in CBD homologs sensitivity. Taken together, these preliminary data show that the three cellular models were sensitive to CBD homologs treatment *in vitro,* despite their different regulation of intracellular signaling pathways.

This is consistent with previous work which cannabinoids affect breast cancer growth with both ER-dependent and -independent mechanisms [20,30].

The microscopic examination of the cells treated with 10 µM CBD homologs revealed massive cytoplasmic vacuolation, especially in MCF-7 cells, a clear signal of cellular stress (Figure S1). A similar effect was observed in mesenchymal MDA-MB-231 cells but in a milder way, while in the epithelial MDA-MB-361 cells no vacuolation occurred (Figure S1). The latter interesting observation needs further investigations that are not included in the present study. In a recent paper similar vacuolar structures were observed in MCF-7 cells treated with a cannabinoid combination: the authors did not determine the origin of these structures but excluded a possible origin from the plasma membrane [31]. They proposed that the cytoplasmic vacuoles could be derived from the ER; they also detected an increased number of lysosomes and the dilation of both ER and mitochondria, resulting in the activation of autophagy and paraptosis pathways. Similarly, Fang W. et al., [32] found that CBD could activate the mitochondrial apoptosis pathway and cause cell damage due to the continuous increase of intracellular ROS. This increase led to the reduction of the mitochondrial transmembrane potential, the opening of the mitochondrial permeability transition pore (mPTP) with subsequent release of cytochrome C into the cytoplasm [33,34], finally resulting in the activation of the mitochondrial-dependent apoptosis pathway. The apoptotic event is preceded by the cell cycle arrest in various cancer models [1,17,35]. Another recent study confirmed that the CBD treatment triggered multiple intracellular effects in MCF-7 cells, such as increased Ca^2+^ levels, ROS accumulation and ER stress, finally leading to the induction of apoptosis [14].

The present study shows that under the tested conditions, all the four CBD homologs exhibited similar behavior, though only the results obtained with the two novel phytocannabinoids have been presented herein. The CBD homologs induced a significant boost of ROS production (Figure 3A), lowering of the mitochondrial functionality (Figure 4), alteration of cell organelles (Figure 5 and Figure 6), ER modification (Figure 7A) and increase in the number of lysosomes (Figure 7B). By the DCFH-DA test the intracellular increase of ROS levels upon CBDB or CBDP treatment was detected (Figure 3C). In addition, the combined treatment of cells with the CBD homologs and NAC evidenced a significant decrease in the relative fluorescence intensity, thanks to the ROS scavengering effect of NAC (Figure 3C). Furthermore, the CBD-driven ROS production is related to the activation of apoptosis, due to impaired mitochondrial function [36], but it is also associated with autophagy [37]. To investigate whether the new CBDB and CBDP led to an impaired mitochondrial function we used two mitochondria stains, Mitotracker Red and MitoTracker Green. In particular, MitoTracker Red is a membrane potential-sensitive dye and is non-fluorescent until entering an actively respiring cell, while MitoTracker Green covalently binds to mitochondrial matrix proteins and allows to monitor the mitochondrial morphology. Figure 4A,B shows that the treatment with both CBDB or CBDB leads to an alteration of the mitochondrial functionality without an appreciable decrease of the mitochondrial mass. The functional loss was also accompanied by a substantial decrease of ATP whose levels dropped by approximately 35–40% in MCF-7 cells treated with all CBD homologs, compared to controls (Figure 4C). A recent study have documented that CBD directly targets mitochondria, revealing multiple dramatic changes in their function and morphology such as swelling and lacking cristae in Jurkat cells [34]. Moreover, the presence of double-membrane vacuoles, containing degrading material (autophagosomes), and the disassembly of Golgi and ER was described. Through the ultrastructural analysis of the CBD homolog-treated cells, we detected multiple damages to cell organelles. First of all, mitochondria with altered morphology were distinguished: in particular the presence of dilated cristae, rounded, rod-like and swollen mitochondria with broken cristae and decreased electron density of the lumen was recognized (Figure 5). TEM imaging also revealed multiple dramatic changes in other cellular compartments. Figure 6B shows the accumulation of double-membrane autophagic vacuoles containing cellular organelles and electron-dense material, while Figure 6C reveals that the ER also appears enlarged and disassembled. This data was confirmed by ER staining with an ER tracker. Indeed, in treated cells, an intense fluorescence signal, likely due to massive ER stress, was detected (Figure 7A). These findings are in accordance with the literature [31]. In addition, the presence of autophagic vesicles in cells exposed to CBD homologs was confirmed through staining with LysoTracker Red, as it is selective for acidic organelles. This optical analysis denotes the accumulation of autophagolysosomes that could be due to an impairment of the autophagic process. Indeed, Shrivastava et al. [20] have shown that CBD may affect the complex cross-talk between autophagy and apoptosis. More recently, Huang et al. reported that CBD, acting via TRPV4, caused mitochondrial dysfunction and lethal mitophagy arrest leading to autophagic cell death in glioma cells [38].

In human cancer cells, the enzyme MAGL plays a major role in the regulation of several processes including cell growth, survival, migration, and invasion [39]. The combination of CBD homologs with drugs that inhibit the activity of enzymes involved in the metabolism of endocannabinoids, such as MAGL inhibitor (MJN110), or with drugs that induce the activation of cellular stress pathways [26,27], such as PMA, is associated with an extensive vacuolization, increased ROS levels, and multiple alterations in cellular organelles, whose effects look more dramatic than those observed when cells were exposed to the CBD homologs alone. Indeed, the combined treatment with CBD homologs and with the drugs (either MJN110 (1 μM) or PMA (100 nM)) induced severe cellular morphological changes, with an increase of about respectively 30% and 60% in the number of cytoplasmic vacuolations (Figure S2). Furthermore, a remarkable rise of ROS levels (Figure 8) and dramatic changes of the organelles structure (Figure S3) were detected in the combined treatment. In the case of PMA treatments, the effects on the cell structures appeared even more catastrophic. In particular, Figure 9 displays a huge number of double-membrane vacuoles containing degrading materials, and the presence of cytoplasmic vacuolations, likely associated with disassembled ER and mitochondria. Further morphological changes at the ER level and an increased number of lysosomes are shown in Figures S4 and S5, respectively. It has been reported that phytocannabinoids exert their action via CB receptor-dependent and independent ways [13,15]. In this regard, it has been also described that mitochondria are the primary CBD target in Jurkat cells [34] and our results are in line with these results.

Hence, CBDB and CBDP are two phytocannabinoids discovered only recently [4,6,7], thus their pharmacological activity is still to be investigated. It is conceivable that both CBDB and CBDP have on the one hand biological properties similar to those of CBD but a different affinity for the target receptors. As for their pharmacokinetics, since CBDB and CBDP are respectively less and more lipophilic than CBD, it is believable that they may have have different absorption rate, metabolism, binding to plasma proteins and elimination rate. However, so far there is no scientific evidence in this sense, and this manuscript represents the first study on the biological activity of these new CBD counterparts

## 4. Materials and Methods

- Cell Culture and Chemicals

The human breast cancer cell lines MCF-7, MDA-MB-361 and MDA-MB-231 were purchased from the American Type Culture Collection (ATCC). These cell lines were maintained in high-glucose Dulbecco’s Modified Eagle’s Medium (DMEM), supplemented with 10% (*v*/*v*) fetal bovine serum, 2% (*v*/*v*) l-glutamine 200 mM and 1% (*v*/*v*) penicillin-streptomycin (5000 U/mL). Cells were maintained in a humidified incubator with 5% CO_2_ at 37 °C. The monoacylglycerol lipase (MAGL) inhibitor, MJN110, was purchased from SIGMA and used at the concentration of 1 µM. *N*-acetyl cysteine (NAC) was purchased from SIGMA and used at 1 mM and 1–0.1 mM, respectively. Nat CBD and syn CBD were kindly provided by CBDepot (Teplice, Czech Republic). Reagents and solvents used in the synthesis were of reagent grade and used without further purification.

- Synthesis and Characterization of CBD Homologs: CBDB and CBDP

CBDB and CBDP were synthesized as reported in previous works [4,7]. Briefly, the synthetic procedure to obtain CBD involved a dropwise addition of a solution of (1*S*,4*R*)-1-methyl-4-(prop-1-en-2-yl) cycloex-2-enol and 5-butylbenzene-1,3-diol (76 mg, 0.50 mmol, 1 eq.) in 5 mL of dry drydichloromethane (DCM) to a solution of 5-butylbenzene-1,3-diol (83 mg, 0.50 mmol, 1eq.) and *p*-toluenesulfonic acid (9 mg, 0.05 mmol, 0.1 eq.) in DCM (5 mL) at −10 °C, under argon atmosphere. The mixture was stirred for 1 h and then quenched with saturated NaHCO_3_ (10 mL). Extraction of the mixture with diethylether (2 × 10 mL) was followed by purification over silica gel (crude:silica gel ratio 1/200, eluent: cyclohexane:DCM 8/2). The chromatographic fractions were analyzed by HPLC-UV and HPLC-HRMS and those containing exclusively CBDB without impurities were collected to give 48 mg of a reddish oil (32% yield, purity > 99%). The same procedure was carried out for the synthesis of CBDP, but 5-heptylbenzene-1,3-diol was used in place of 5-butylbenzene-1,3-diol to obtain a linear heptyl side chain. CBDP was obtained with a 23% yield (76 mg) as a colorless oil (purity > 99%).

The purity of CBDB and CBDP was checked by HPLC-HRMS analysis using an Ultimate 3000 liquid chromatograph (Thermo Fisher Scientific, Grand Island, NY, USA), equipped with a vacuum degasser, a binary pump, a thermostated autosampler, and a thermostated column compartment. The chromatographic system was interfaced to a heated electrospray ionization source and a Q-Exactive Orbitrap mass spectrometer (HPLC-HRMS). The chromatographic separation was carried out on a Poroshell 120 SB-C18 (3.0 × 100 mm, 2.7 μm, Agilent, Milan, Italy). The same instrumental parameters used in previous works were applied to confirm the identity of the synthesized compounds [4,7]. In detail, an isocratic elution of 30% water with 0.1% formic acid (A) and 70% acetonitrile (ACN) with 0.1% of formic acid (B) was set for 10 min, then 95% B was pumped for 5 min and lastly, the column was re-equilibrated for 2 min with the initial conditions for a total run time of 17 min. The flow rate was maintained constant at 0.5 mL/min. 5 µL of 0.1 µg/mL solutions of CBDB and CBDP were separately injected into the analytical system. The parameters of the heated electrospray ionization source were set as follows: capillary temperature, 320 °C; vaporizer temperature, 280 °C; electrospray voltage, 4.2 kV for positive mode and 3.8 kV for negative mode; sheath gas, 55 arbitrary units; auxiliary gas, 30 arbitrary units; S lens RF level, 45. Analyses were acquired using the Xcalibur 3.0 software (Thermo Fisher Scientific, San Jose, CA, USA) in full scan data-dependent acquisition (FS-dd-MS^2^) in positive and negative mode at a resolving power of 70,000 FWHM at *m*/*z* 200. The parameters of the Orbitrap mass analyzer were as follows: scan range of *m*/*z* 250–400, AGC of 3e6, injection time 100 ms, and isolation window for the filtration of the precursor ions of *m*/*z* 0.7. Normalized collision energy (NCE) of 20 was used to fragment the precursor ions. Extracted ion chromatograms (EIC) of the [M + H]+ and [M − H]− molecular ions were derived from the total ion chromatogram with a 5-ppm mass tolerance.

- Western Immunoblot Analysis

Cells were lysed in a RIPA buffer (Cell Signaling) supplemented with protease inhibitors Cocktail (1×) and sodium fluoride (NaF, 16 µL/mL). Protein concentration was determined by the Bradford protein assay (BIO-RAD, Hercules, CA, USA). Samples were mixed 1:1 with Laemmli buffer (SIGMA, St. Louis, MO, USA), boiled for 5 min, 90 °C and 25 μg of proteins were separated onto Mini-PROTEAN^®^ TGX™ Precast Gels (BIO-RAD, Hercules, CA, USA). Electrophoresis was run at 200 V for 60 min (IEF Cell Protean System, BIO-RAD, Hercules, CA, USA) and consequently total protein bands were visualized by 2.5 min of UV exposure, for gel activation. The bands were then transferred to the Midi Nitrocellulose membrane Trans-Blot Turbo (BIO-RAD, Hercules, CA, USA). The membranes were blocked for 1 h in Blotto A (Santa Cruz, CA, USA) at room temperature and subsequently probed for 1 h by the appropriately diluted primary antibodies. After three washes with a solution containing 10 mM Tris, pH 8.0, 150 mM NaCl, 0.5% Tween 20 (TBST solution), blots were incubated with secondary antibody HRP-conjugated for 1 h at room temperature (1:2000 dilution). Blots were then developed using the Clarity Enhanced chemiluminescence (ECL) (BIO-RAD, Hercules, CA, USA). Primary antibodies (1:1000 dilution) were: from Santa Cruz CB1 (2F9) sc-293419, CB2 (3C7) sc-293188 and from cell signaling p38 MAPK (#8690). Secondary antibodies (HRP-conjugated) were from Bethyl Laboratories (1:5000 dilution) (mouse IgG-heavy and light chain antibody, A90-116P). Images shown in the paper are representative of three independent replicates. Densitometric quantification of the band intensity was normalized to p38 levels using ImageJ.

- Cell Viability Assay

Changes in viability after the various treatments were measured using the Thiazolyl blue tetrazolium bromide (MTT) assay. Cells were seeded in 96-well plates at a density of 1 × 10^4^/well and incubated at 37 °C in 5% CO_2_. After overnight incubation, the medium was replaced with vehicle control or drug at different concentrations in DMEM and supplemented with 10% (*v*/*v*) fetal bovine serum, 2% (*v*/*v*) L-glutamine 200 mM and 1% (*v*/*v*) penicillin-streptomycin (5,000 U/mL). The cell lines were maintained in a humidified incubator with 5% CO_2_ at 37 °C. After 24 h, upon completion of the drug treatments, the medium was removed and replaced with a serum-free medium containing 2 mg/mL MTT and incubated for 2 h at 37 °C. The MTT reagent was then removed and the formazan crystals were solubilized using dimethyl sulfoxide. The absorbance was read using the CLARIO star Plus microplate reader (570 nm). The absorbance of the vehicle control was subtracted and the percentage control was calculated as the absorbance of the treated cells/control cells × 100.

- Measurement of Reactive Oxygen Species (ROS)

To detect the changes in intracellular ROS levels, 2′,7′- dichlorofluorescein diacetate (DCFH-DA, Sigma-Aldrich) staining was used (Hyeoncheol Kim et al.). DCFH-DA is a stable, fluorogenic and non-polar compound that can readily diffuse into the cells and get deacetylated by intracellular esterases to a non-fluorescent 2′,7′- dichlorodihydrofluorescein (DCFH) which is later oxidized by intracellular ROS into highly fluorescent 2′,7′-dichlorofluorescein (DCF). The intensity of fluorescence is proportional to intracellular ROS levels. MCF-7 cells were seeded at a density of 2 × 10^5^ cells per well in 24 well plates and were allowed to attach overnight. On the first day of treatment, the medium was replaced with the fresh ones containing the vehicle control, CBD homologs (10 µM), NAC (1 mM), PMA (1 µM) and MJN110 (1 µM) respectively. After 24 h, upon completion of the drug treatment, the spent medium was removed. The cells were washed once with fresh DMEM, and twice with 1X PBS and incubated with DCFH-DA in a final concentration of 10 µM for 30 min. Cells were rinsed with PBS and representative fluorescent images for each well using the green fluorescent protein (GFP) channel on an Evos m7000 fluorescence microscope were taken. After taking images, PBS was removed and a radioimmunoprecipitation assay (RIPA) buffer was added to each well. The collected cells were incubated at −80 °C for 20 min and then centrifuged at 21,130× *g* for 10 min at 4 °C. The collected supernatant was transferred to a black 96 well plate and the fluorescence intensity measured using the CLARIO star Plus microplate reader at an excitation wavelength of 485 nm and an emission wavelength of 530 nm. After fluorescence recording, 5 µL of supernatant were transferred to a clear 96 well plate containing 195 μL of 1× protein assay solution to measure the protein concentration using the BCA assay. The fluorescence intensity was normalized to the protein concentration.

- MitoTracker Staining

The determination of the mitochondrial membrane potential was performed with the Mitotracker assay. MCF-7 cells were seeded at a density of 2 × 10^5^ cells per well in a 24-well plate and were allowed to attach overnight, 37 °C. On the first day of treatment, the medium was replaced with vehicle control or CBD homologs (10 µM) in DMEM. After 24 h, upon completion of drug treatments, the spent medium was removed. The cells were then washed with fresh DMEM prior to incubating them with 75 nM of either MitoTracker™ Green FM or MitoTracker™ Red CMXRos in pre-warmed medium, without FBS, for 30 min, at 37 °C. Then the medium was removed, the cells were rinsed with PBS and representative fluorescent images for each well using respectively the GFP and the RFP channel on an Evos m7000 fluorescence microscope were taken.

- CellTiter-Glo 2.0 Cell Viability Assay

The quantification of the cellular ATP was performed using the CellTiter-Glo 2.0 cell viability assay. Cells were seeded in 96-well plates at a density of 1 × 10^4^ /well and incubated at 37 °C in 5% CO_2_. After overnight incubation, the medium was replaced with either vehicle control or drug at different concentrations in DMEM and supplemented with 10% (*v*/*v*) fetal bovine serum, 2% (*v*/*v*) L-glutamine 200 mM and 1% (*v*/*v*) penicillin-streptomycin (5000 U/mL). The cell lines were maintained in a humidified incubator with 5% CO_2_, at 37 °C. After 24 h, upon completion of the drug treatments, the CellTiter-Glo 2.0 reagent was added into each well at the equivalent volume of cell culture medium in the well. Then, the contents were mixed vigorously for 5 min to induce cell lysis, and the plate was incubated at room temperature for an additional 25 min to stabilize the luminescent signal. Afterward, the supernatants were transferred in technical replicates into the 96-well opaque white-walled plate and the luminescence was measured using the CLARIO star Plus microplate reader.

- Ultrastructural Analysis of the Cellular Samples

MCF-7 cells were seeded at a density of 5 × 10^5^ cells in Primo TC flasks 25 cm^2^ and were allowed to attach overnight. Control and CBD homologs treated cells were grown to 80% confluency and fixed with 2.5% glutaraldehyde in 0.1 M cacodylate buffer, pH 7.4, for 2 h at 4 °C. The cells were washed three times, 5–10 min each, in ice-cold PBS buffer and then post-fixed in ice-cold 1% osmium tetroxide. After 1 h, the samples were washed three times in PBS and dehydrated in an acetone series for 15 min each with 25, 50, 75, 90 and 100% acetone. Three steps of infiltration in a mixture of resin/acetone (1/2, 1/1 and 2/1 ratios) were performed and finally, the specimens were embedded in 100% resin at 60 °C for 48 h. Ultrathin sections (70 nm thick) were cut with an Ultramicrotome. TEM images were recorded on a JEOL Jem1011 microscope operating at an accelerating voltage of 100 kV (Tokyo, Japan).

- Lysotracker Assay

The mitochondrial-lysosomal axis theory of aging postulates that oxidized material accumulates in lysosomes as cells age, which results in a decreased degradative capacity of lysosomes. This behavior was studied using Lysotracker staining. MCF-7 cells are seeded at a density of 2 × 10^5^ cells per well in a 24-well plate and are allowed to attach overnight, at 37 °C. On the first day of treatment, the medium was replaced with vehicle control, CBD homologs (10 µM), NAC (1 mM), PMA (1 µM) and MJN110 (1 µM) in DMEM. After 24 h, upon completion of the drug treatments, spent media was removed. The cells were washed once with fresh DMEM. Cells were incubated with HOECHST (0.5 mg/mL in PBS), for 5 min, at 37 °C. The Hoechst was removed and replaced with a pre-warmed medium, without FBS, containing 100 nM LysoTracker Red DND-99 (Cat. No. L-7528). Cells are incubated at 33 °C for 30 min with LysoTracker. Then the medium was removed, the cells were rinsed with PBS and representative fluorescent images for each well using the RFP channel on an Evos m7000 fluorescence microscope were taken.

- Endoplasmic Reticulum Staining

MCF-7 cells are seeded at a density of 2 × 10^5^ cells per well in a 24-well plate and are allowed to attach overnight, at 37 °C. On the first day of treatment, the medium was replaced with vehicle control, CBD homologs (10 µM), NAC (1 mM), PMA (1 µM) and MJN110 (1 µM) in DMEM. After 24 h, upon completion of the drug treatments, spent media was removed. The cells were washed once with fresh DMEM. Cells were stained with ER-Tracker™ Green (glibenclamide BODIPY^®^ FL), 1 µM in pre-warmed medium, without FBS for 15 min at 37 °C. Then the medium was removed, the cells were rinsed with PBS and representative fluorescent images for each well using the GFP channel on an Evos m7000 fluorescence microscope were taken.

## 5. Conclusions

Data collected in this study suggest that the treatment of MCF-7 cells with CBDB and CBDP activates catastrophic intracellular processes. Though the newly discovered CBDB and CBDP differ from the well-known nat CBD and syn CBD only for the length of the alkyl side chain on the resorcinic portion, their biological effects look comparable to those observed in cancer cells exposed to nat and syn CBD. Additionally, our results provide evidence that CBD homologs alter the morphology and the structure of multiple organelles, affecting cell homeostasis. These preliminary results represent the first step of further in-depth studies required to confirm the potential use of these homologs as an adjuvant in anticancer chemotherapy.

## Figures and Tables

**Table 1:** IC_50_ values (µM) are reported as mean ± S.D. of three independent experiments (*n* = 3). MCF-7, MDA-MB-361 and MDA-MB-231 cells were treated with nat CBD, syn CBD, CBDB and CBDP (from 0 to 100 µM) for 24 h.

Compounds	*MCF-7*	*MDA-MB-361*	*MDA-MB-231*
*NAT CBD*	58.6 ± 0.1	49.8 ± 1.5	46.6 ± 1.0
*SYN CBD*	59.0 ± 1.2	53.2 ± 0.8	45.3 ± 0.9
*CBDB*	57.4 ± 1.2	56.4 ± 1.2	48.4 ± 0.6
*CBDP*	56.7 ± 0.4	60.9 ± 0.4	49.3 ± 1.4

## Supplementary Materials

The following are available online. Figure S1: Representative images of MCF-7, MDA-MB-361 and MDA-MB-231 cells treated with cannabinoid homologs, Figure S2: Quantification of vacuolated cells and representative images of MCF-7 cells treated with either MJN110 or PMA, Figure S3: Representative TEM images of cell morphology, Figure S4: Representative fluorescent images of organelles structures from MCF-7 cells treated with MJN110, Figure S5: Representative fluorescent images of organelles structures from MCF-7 cells treated with PMA.
