# Supplementary material for: Oxidative Stress and Multi-Organel Damage Induced by Two Novel Phytocannabinoids, CBDB and CBDP, in Breast Cancer Cells
Source: Molecules. 2021 Sep 14;26(18):5576. doi: 10.3390/molecules26185576 (PMC8467640; doi:10.3390/molecules26185576)
Supplement: Supplementary file 1 [file molecules-26-05576-s001.zip › molecules-1354797-supplementary.pdf]

molecules

MDPI

Supplementary Materials:

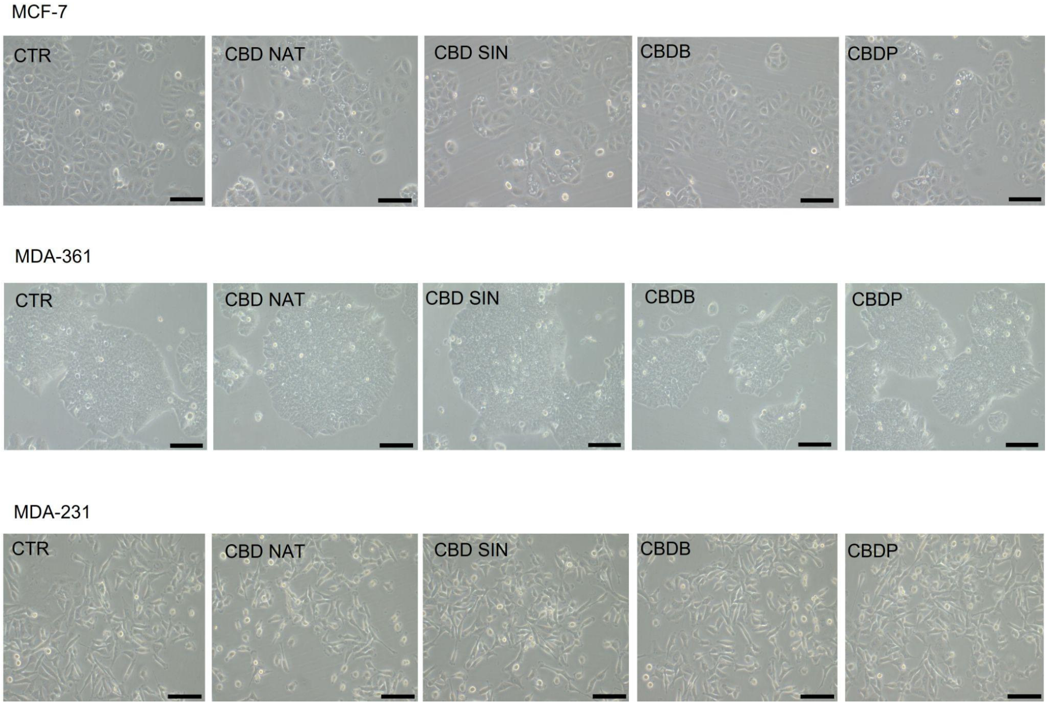

S1: Representative images of MCF-7, MDA-MB-361 and MDA-MB-231 cells treated with vehicle (DMSO) or cannabinoid homologs at the concentration of 10 µM for 24 h. Images were acquired using an inverted wide-field microscope (Olympus IX51), objective 10x. Scale bar corresponds to 100 µm.

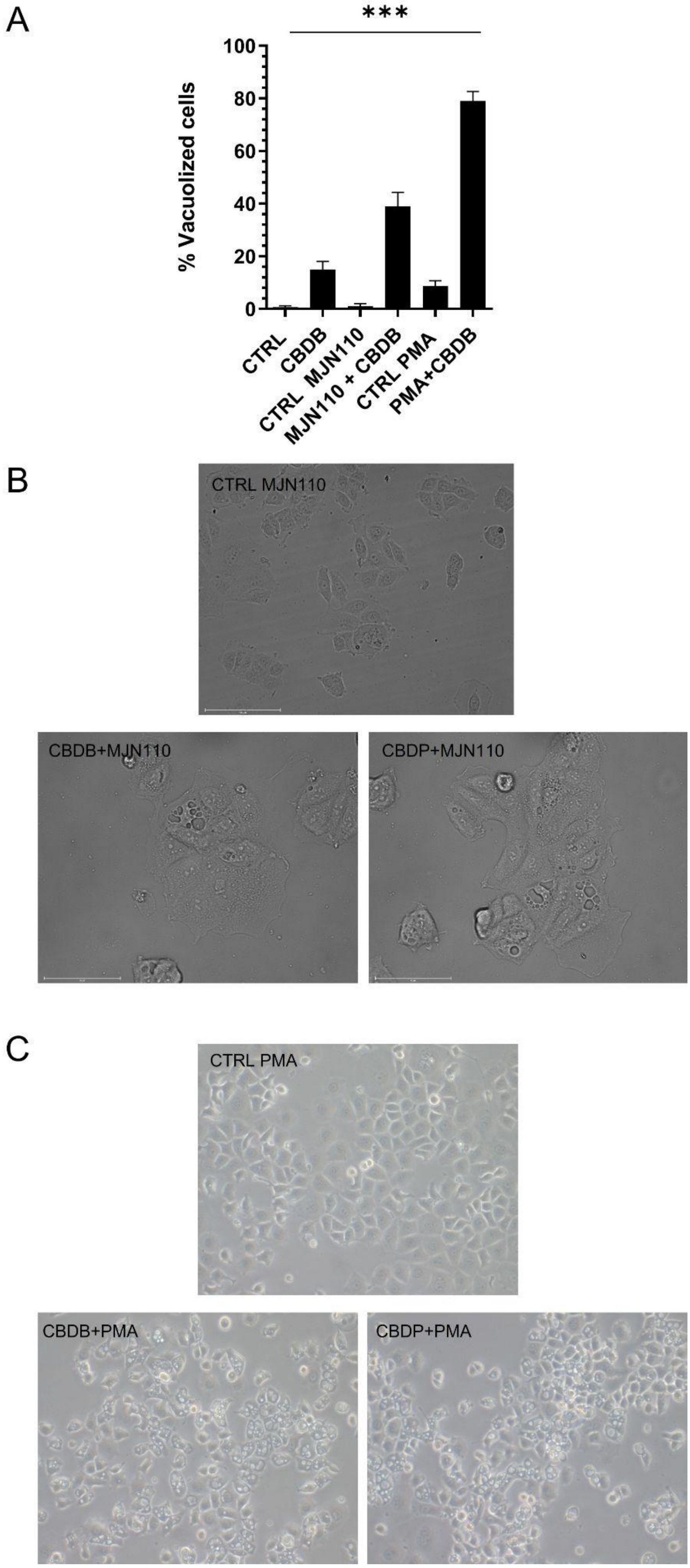
A
***
100
80
60
40
20
0
% Vacuolized cells
CTRL
CBDB
CTRL MJN110
MJN110 + CBDB
CTRL PMA
PMA+CBDB
B
CTRL MJN110
CBDB+MJN110
CBDP+MJN110
C
CTRL PMA
CBDB+PMA
CBDP+PMA

S2: (A) Quantification of vacuolated cells and average vacuole sizes in the presence or absence of the indicated treatments. Values are the mean ± SD. p-value **** < 0.0001, **P < 0.01; ****P < 0.0001 compared with control; (B) Representative images of MCF-7 cells treated with vehicle (DMSO+MJN110), CBDB or CBDP (10 μM) in combination with MJN110 (1 μM) for 24 h. Images were acquired using an Evos m7000 fluorescence microscope. Scale bar 150 μm;), objective 20x (C) Representative images of MCF-7 cells treated with vehicle (DMSO+PMA), CBDB or CBDP (10 μM) in combination with PMA (1 μM) for 24 h. Images were acquired using an inverted wide-field microscope (Olympus IX51). Scale bar 100 μm,), objective 10x.

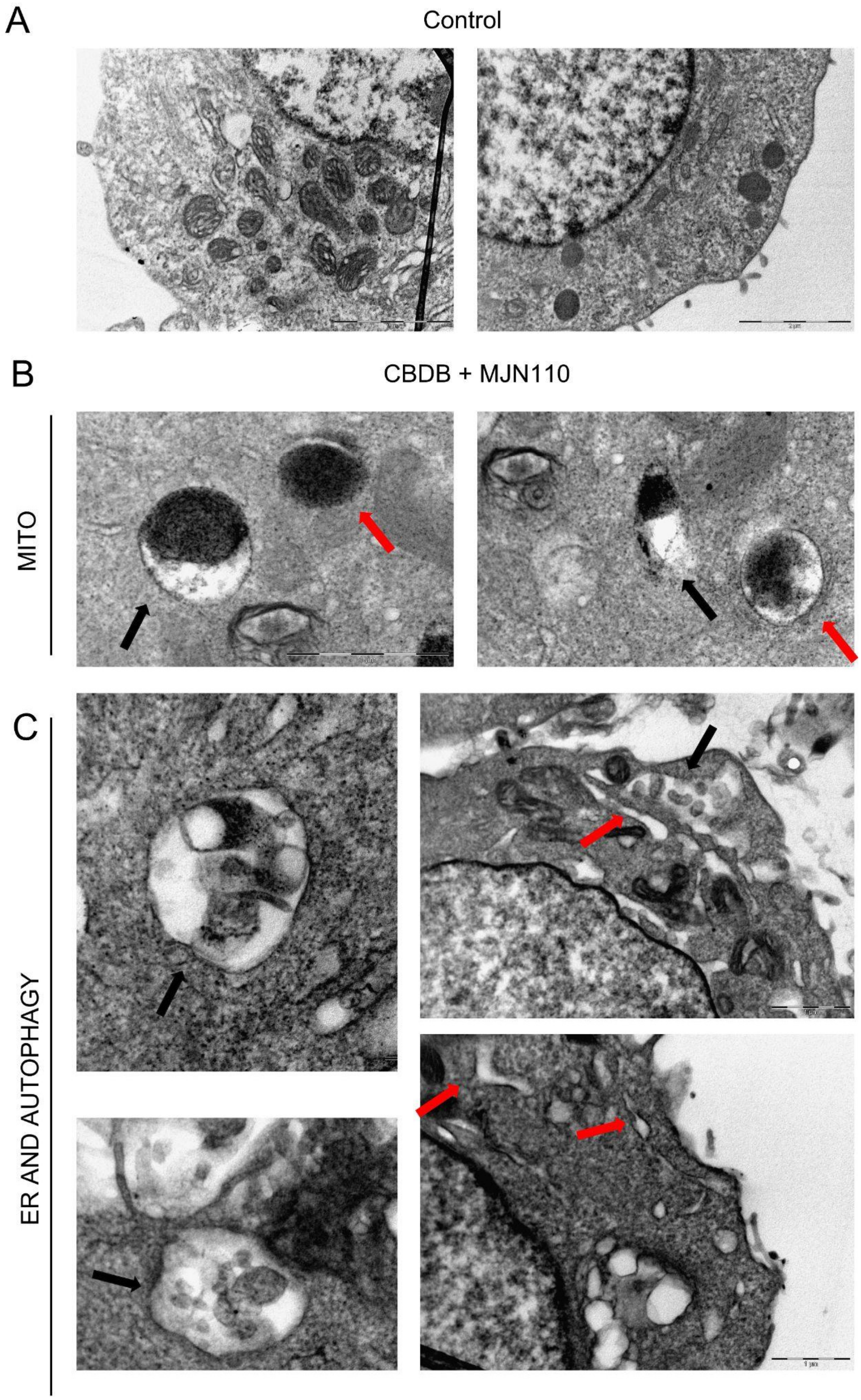

S3: Representative TEM images of cell morphology. (A) MCF-7 cells are treated with vehicle (DMSO+MJN110) as controls showing normal cell organelles. Scale bar 2 μm. (B) MCF-7 cells fixed after incubation with CBDB + MJN110 (1 μM), mitochondria with dilated cristae (black arrow) and swollen mitochondria with decreased electron matrix density (red arrow); (C) autophagic vacuoles containing different organelles or electron-dense material (black arrows); outstretched ER (red arrows). Scale bar 1 μm.

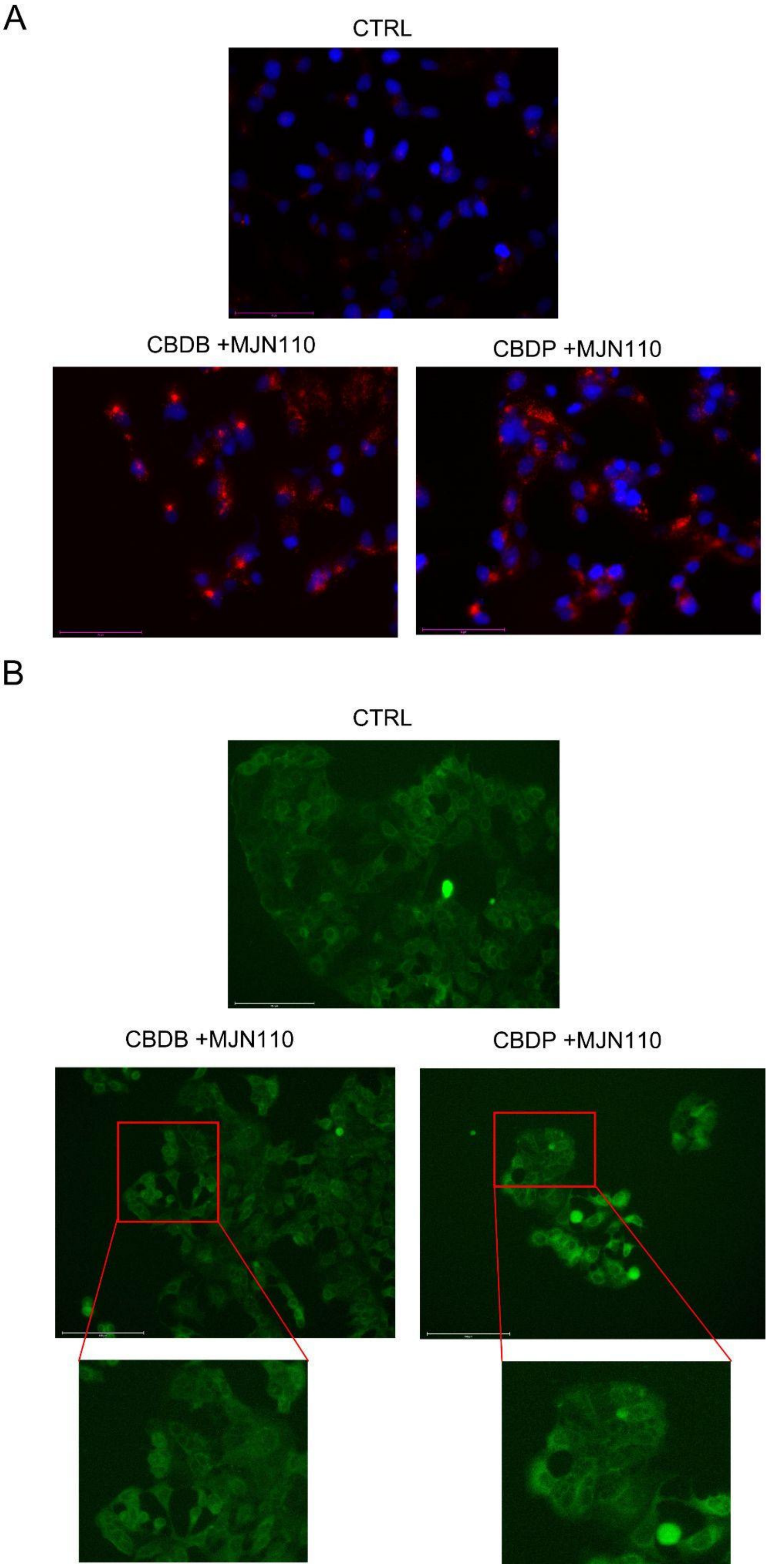
A
CTRL
CBDB +MJN110
CBDP +MJN110
B
CTRL
CBDB +MJN110
CBDP +MJN110

S4: Representative fluorescent images of organelles structures. MCF-7 cells are treated with vehicle (DMSO+MJN110) and MJN110+ CBDB and CBDP for 24 h. (A) Cells were stained with the Hoechst (0,5 mg/mL) and Lysotracker Red DND-99 (100 nM); (B) Cells were stained with ER TrackerTM to visualize the endoplasmic reticulum membrane according to manufacturer's recommendations and imaged with an Evos m7000 fluorescence microscope. Scale bar 150 μm,), objective 20x.

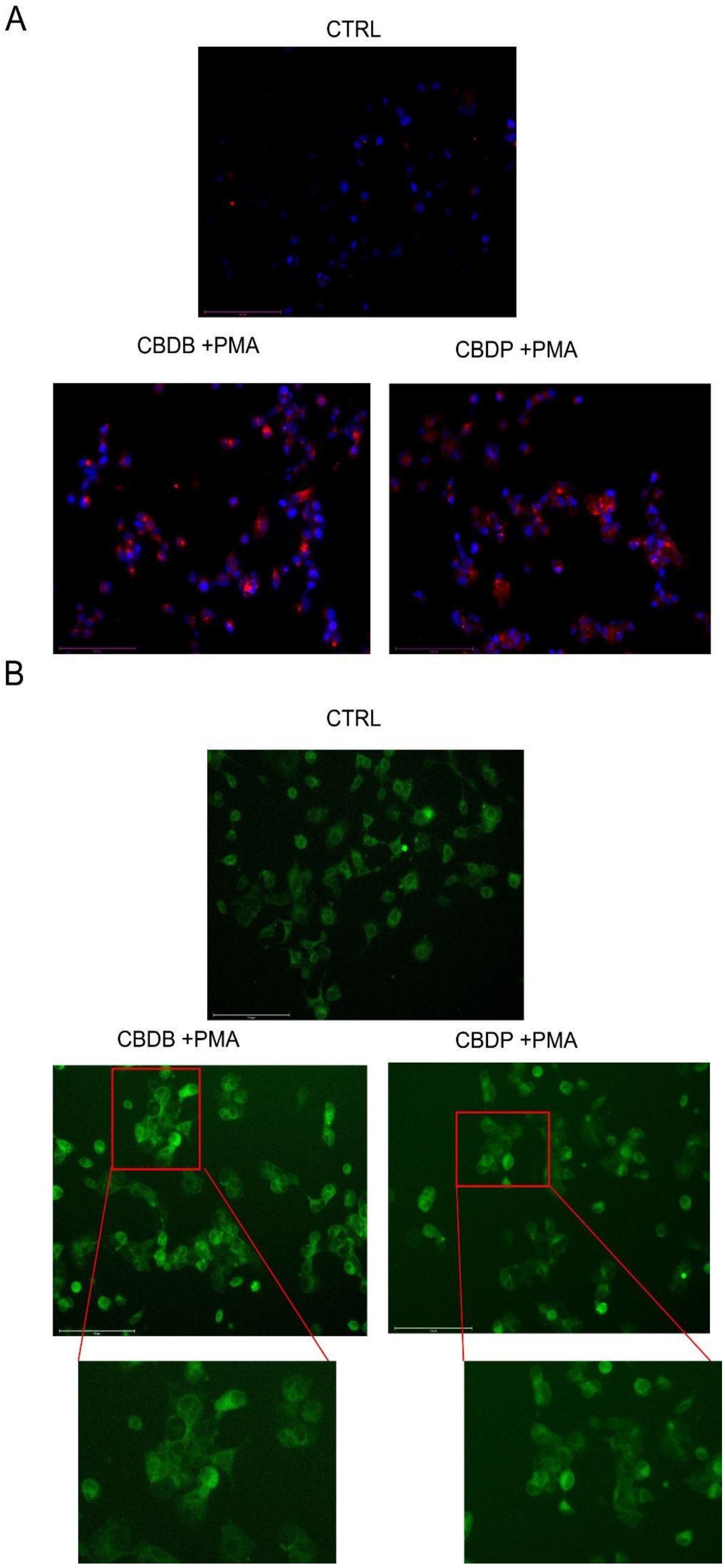

S5. Representative fluorescent images of organelles structures. MCF-7 cells are treated with vehicle (DMSO+PMA) and PMA + CBDB and CBDP for 24 h. (A) Cells were stained with the Hoechst (0,5 mg/mL) and Lysotracker Red DND-99 (100 nM); (B) Cells were stained with ER TrackerTM to visualize the endoplasmic reticulum membrane according to manufacturer's recommendations and imaged with an Evos m7000 fluorescence microscope. Scale bar 150 μm,), objective 20x.
